# Biocatalytic conversion of microalgal biomass to biodiesel: optimization of growth conditions and synthesis of CaO bionanocatalyst from *Monoraphidium* sp. NCIM 5585

**DOI:** 10.1038/s41598-025-88792-w

**Published:** 2025-02-05

**Authors:** Supriya Pandey, Thivaharan Varadavenkatesan, Raja Selvaraj, Ramesh Vinayagam

**Affiliations:** 1 Department of Biotechnology, Manipal Institute of Technology, Manipal Academy of Higher Education, Manipal, 576104 Karnataka India; 2 Department of Chemical Engineering, Manipal Institute of Technology, Manipal Academy of Higher Education, Manipal, 576104 Karnataka India

**Keywords:** Microalgae, Biodiesel, Bionanocatalyst, Light intensity, Photoperiod, Nitrogen stress, Biotechnology, Nanoscale materials

## Abstract

Microalgal feedstock is a potential source for biodiesel production that addresses the challenges of fuel security and sustainable agriculture. This study aims to maximize biomass yield and lipid accumulation for freshwater microalga *Monoraphidium* sp. NCIM 5585 and utilize it for biodiesel production, contributing to the development of biocatalysis-based biofuels. Independent optimization studies were conducted to investigate critical growth parameters, viz., light intensity, photoperiod, and NaNO_3_ concentration. The study showed highest biomass productivity of 51.75 ± 1.9 mg/L.d and lipid content of 47.3 ± 0.02% (w/w) at 40 µmol/m^2^/s light intensity, 16 h L:08 h D photoperiod, and 0.25 g/L NaNO_3_. Further, a novel CaO bionanocatalyst was synthesized using residual microalgal biomass and characterized using SEM, EDX, FT-IR, and XRD. The characterization results from SEM and EDX confirmed the structural and elemental composition of bionanocatalyst with Ca and O as main elements. XRD revealed the crystalline nature of CaO with particle size of 17.83 nm. 86.5 ± 0.65% (w/w) FAME was obtained using the synthesized catalyst and was characterized using ^1^H NMR, ^13^C NMR and GC-MS. This study demonstrates the potential of *Monoraphidium* sp., optimized growth conditions and the significance of reusability of residual microalgal biomass as catalyst for sustainable biodiesel production, offering a promising solution for fuel security and biotechnology applications.

## Introduction

Growing energy demands have increased the research interest to especially cater to the transportation fuel requirements. Conventional fossil fuel reserves are finite and contribute significantly to environmental degradation. The accelerating pace of industrialization and globalization underscores the urgent need to explore and adopt renewable and sustainable energy sources, such as biofuels. Biofuels produced from microalgal feedstock offer a cleaner alternative by mitigating environmental pollution and production cost. Microalgae are highly promising resources offering diverse energy and environmental benefits, including bioenergy production, nutrient recovery, and carbon sequestration. Microalgae as well as macroalgae are used for the production of not only biodiesel but other crucial biofuels such as bio-ethanol, bio-methanol, bio-hydrogen and bio-oil^1^. Biodiesel is among the most extensively studied biofuels, emerging as a viable alternative to fossil-based diesel^2^. Its potential to decrease reliance on conventional diesel and lower pollution emissions has garnered significant scientific interest. With carbon effectively sequestered in its exhaust, biodiesel exhibits net-zero carbon emissions. Moreover, its use does not contribute to an increase in atmospheric carbon dioxide (CO₂), thereby helping to mitigate the greenhouse effect^3^.

Biodiesel produced from microalgal feedstock is renewable, sulfur-free, and non-toxic^4^. It is a long chain of fatty acid monoalkyl esters (FAMEs) produced via transesterification of neutral lipids obtained from microalgae. Microalgae are single-celled photosynthetic organisms and so deemed to be the evolutionary predecessors of plants^5^. They inhabit both freshwater and marine water habitats. They synthesize and store proteins, carbohydrates, lipids, and pigments such as carotenoids. The neutral lipid fraction accumulated in them accounts for the promise they hold towards biodiesel production^6^. Freshwater microalgae are preferred because they are easy to cultivate in limited space, thus resulting in reduced competition for land availability^7^.

Microalgae such as *Ettlia* sp., *Monoraphidium* sp., *Chlorella vulgaris*, and *Nannochloropsis oceanica* have been reported with 10–30% of lipid content. The current study discusses *Monoraphidium* sp., a freshwater green microalga, taxonomically categorized within the *Selenastraceae* family. It is characterized by elongated cells that exhibit straight/crescent/bean shapes^8^ and is known for biodiesel production. Numerous microalgal species are known to accumulate substantial lipid content within their cells, making them promising candidates for lipid production. These lipids can be efficiently converted into biodiesel through transesterification, highlighting the potential of microalgae as a sustainable alternative source of bioenergy for the future^1^. Microalgae accumulate lipids as storage lipids mainly in the form of triacylglycerides (TAGs), so only a small quantity of lipids is synthesized under optimized cultivation conditions. Synthesis and breakdown of TAGs in microalgae are linked to stress-induced metabolism^9^. Exposure to extreme cultivation conditions and modifying the nutritional regimes force the microalgae to accumulate lipids as part of its self-defense mechanism against photooxidation^10^.

Environmental factors such as light intensity, pH, temperature, aeration, and composition of growth media influence both biomass and lipid productivity^11^. Nutrient (nitrogen and phosphorus) limitation or exposure to prolonged photoperiod and high light intensities are highly influential factors^12^. In this direction, various microalgae have been studied for biodiesel production, but very few discuss growth and optimization under the autotrophic mode of cultivation. Under the autotrophic mode of cultivation, the optimization of such parameters can be studied well as there is a low risk of contamination and growth variation. It has been studied that the combined effect of two or more stress conditions increases the rate of lipid accumulation. Nitrogen stress (via NaNO_3_ concentration), high light intensity (> 600 µmol/m^2^/s), and prolonged photoperiod 20 h L: 04 h D have been reported for various freshwater microalgae such as *Scenedesmus* sp. *Chlorella* sp, and *Chlorococcum* sp^13^. Shen et al. found that stress conditions induce lipid accumulation in *C. vulgaris*^4^. Under stress, the cellular metabolism of microalgae is reduced which results in the synthesis of secondary metabolites. The lipid productivity increases but the cell growth is reduced. However, the response to stress conditions varies from species to species^13^. Thus, the growth conditions best suited for high biomass and lipid productivity should be optimized for individual species in a defined culture medium. This study aims to determine the ideal optimized growth condition for *Monoraphidium* sp. NCIM 5585. Under such conditions, the microalgae are cultivated and then followed by biomass harvesting and cell disruption. Cell disruption using chemical or mechanical methods aids maximum extraction of lipids from the cells. Lipid extraction using the Bligh and Dyer method has been widely reported and used to quantify lipid productivity^14^.

Lipid-to-biodiesel conversion requires TAG derivates and an alcohol (methanol/ethanol), where the alcoholic group replaces the glycerol in the fatty acid chain forming fatty acid alkyl esters (FAAEs) or biodiesel. This transesterification reaction is mediated by a catalyst, homogenous or heterogeneous, acidic or basic. Several bionanocatalysts derived from various biological sources such as eggshells, plant extracts, and lipases extracted from fungi have been used as catalysts for transesterification reactions. Algae, like plants, are rich sources of secondary metabolites and hence facilitate the reduction and stabilization of the metal to form nanoparticles^15^. CaO catalyst has been synthesized using animal shells and plant extracts. It has been reported to successfully transesterify microalgal lipids to biodiesel^16^. Further, macroalgal species such as *Ulva lactuca* and *Sargassum plagiophyllum*, microalgae such as *(A) dimorphus*, *C. vulgaris*, and *(B) braunii* have been reported to be used for fabricating bionanocatalysts using various nanoparticles^17^. However, the synthesis and usage of microalgae-based nanoparticles have scarcely been reported to mediate microalgal lipid transesterification. Thus, to overcome this research gap current study highlights upon the importance of synthesis and utilization of microalgae-based nanoparticles (bionanocatalyst). This study aims to synthesize a novel bionanocatalyst which is sustainable and cost-effective using the residual biomass of *Monoraphidium* sp. NCIM 5585 and then utilize the same for mediating the transesterification reaction. Thus, the objectives of the study are to address the importance of growth condition optimization for microalgae to enhance biomass and lipid productivity, the fate of residual microalgal biomass, the significant measures for catalyst cost reduction (via synthesis, characterization and utilization of CaO bionanocatalyst in transesterification process) and the catalyst’s influence on biodiesel production.

## Materials and methods

### Materials

Chemicals used in the study, viz., ethanol, trichloroacetic acid, Bradford reagent, acetone, vanillin, 85% ortho-phosphoric acid, 98% sulphuric acid, oleic acid, methanol, and chloroform were obtained from M/s Merck Ltd, Mumbai. Calcium nitrate and anthrone were obtained from Loba Chemie. Sodium hydroxide was purchased from M/s SRL Pvt Ltd, Mumbai.

### Microalgae culture and maintenance conditions

**Fig. 1:**
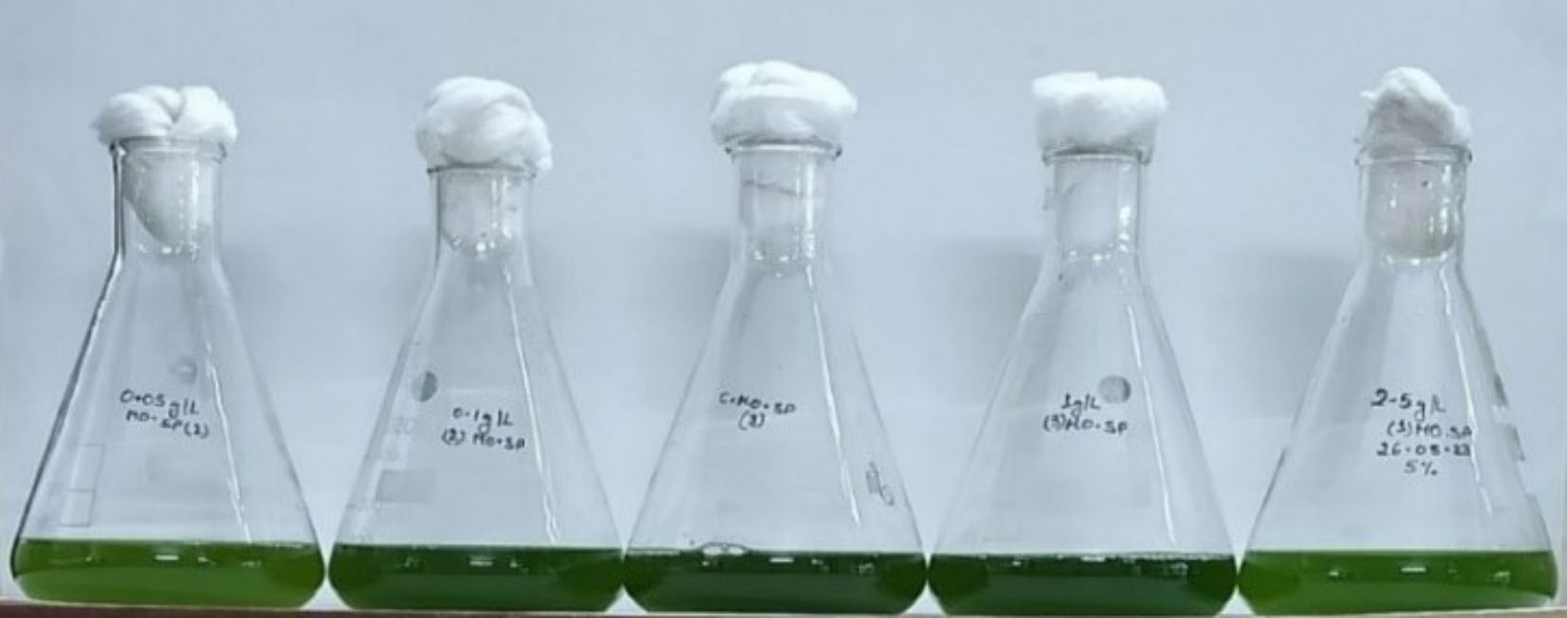
Cultivation of *Monoraphidium* sp. NCIM 5585.

The microalgal strain, viz.,* Monoraphidium* sp. NCIM 5585 (MO) was obtained from the National Collection for Industrial Microorganisms (NCIM), Pune, Maharashtra, India. MO was cultivated autotrophically using Bold’s Basal Medium (BBM). The medium consisted of (g/L): NaNO_3_ (25), K_2_HPO_4_ (7.5), KH_2_PO_4_ (17.5), NaCl (2.5), MgSO_4_.7H_2_O (7.5), CaCl_2_.2H_2_O (2.5), alkaline EDTA solution (EDTA – 50 and KOH – 31), H_3_BO_3_ solution (11.42), acidified iron solution (H_2_SO_4_ – 1mL; FeSO_4_.7H_2_O – 4.98) and trace element solution (MnCl_2_.4H_2_O – 1.44; ZnSO_4_.7H_2_O – 8.82; CuSO_4_.5H_2_O – 1.57; Co(NO_3_)_2_.6H_2_O – 0.49)^18^. All the components were constituted in distilled water. Sterilized BBM (250 mL) was inoculated with 10% (v/v) MO inoculum. The standard cultivation conditions, viz., 40 µmol/m^2^/s light intensity, 10 h L: 14 h D photoperiod cycle, 25–28 ºC, 7–8 pH were maintained for 30 days, with no external aeration (Fig. 1). Growth was monitored by cell counting method using a hemocytometer.

### Biochemical characterization

Carbohydrate estimation was carried out using the anthrone method. 100 mg of dried MO biomass was homogenized in a mortar using a pestle and 80% ethanol. The obtained homogenate was centrifuged for 20 min at 4000 rpm. 1 mL of supernatant mixture and 2 mL of anthrone reagent were incubated in a water bath for 10 min at 100 ºC. The optical density was measured at 620 nm^19^. The total protein content was estimated by the Bradford method. 100 mg of the dried MO biomass was homogenized in a mortar using a pestle and 2 mL of 20% trichloroacetic acid (TCA). The homogenate was centrifuged for 15 min at 8000 rpm. The obtained pellet was suspended in 5 mL of 0.1% NaOH, re-centrifuged and the supernatant was collected. 1 mL of the supernatant and 1 mL of the Bradford reagent were added into a test tube and incubated in the dark at room temperature for 10 min. Absorbance was recorded at 595 nm^20^. 100 mg of the dried MO biomass was homogenized in a pre-cooled mortar using a pestle and 80% (v/v) acetone. The extract obtained was centrifuged at 3000 rpm for 15 min. The optical density of the supernatant was measured at 645 nm and 663 nm to determine the levels of chlorophyll ‘a’, chlorophyll ‘b’, and total chlorophyll^21^.

### Optimization of growth condition

Three physical parameters, viz., photoperiod, light intensity, and NaNO_3_ concentration were chosen as the parameters to optimize the growth of MO. All the flasks were maintained at a temperature between 25 and 28 ºC and pH in the range 7–8. In the investigation studying the effect of the three parameters, the control (C) flask was maintained at a photoperiod of 10 h L:14 h D, 40 µmol/m^2^/s light intensity, and 0.25 g/L NaNO_3_ concentration. In the batch of experiments studying the effect of light intensity, it was pegged at 20, 60, and 100 µmol/m^2^/s with the photoperiod held at 10 h L:14 h D and NaNO_3_ concentration at 0.25 g/L. With light intensity and NaNO_3_ concentration fixed at 40 µmol/m^2^/s and 0.25 g/L respectively, the effect of photoperiod was studied by varying it at 12 h L:12 h D, 16 h L: 08 h D and 20 h L: 04 h D cycle. NaNO_3_ concentration was fixed at 0.05, 0.1, 0.25, 1, 2.5 g/L in the BBM medium without changing the composition of other medium components. Light intensity and photoperiod were fixed at 40 µmol/m^2^/s and 10 h:14 h (L: D) respectively.

### Determination of growth parameters

#### Cell growth

Growth of the MO strain was monitored using a hemocytometer for 30 days. 1 mL of the culture sample was loaded on the slide chamber. The slide was placed under a microscope and cells were counted in the Neubauer chamber. Cell concentration and specific growth rate were determined^22^.

#### Dry weight of biomass

The weight of empty vials was initially recorded, and 10 mL of each culture sample was added to it. The samples were centrifuged at 10,000 rpm for 15 min to obtain the microalgal biomass pellet. The supernatant was discarded, and the biomass, was retained in the vial. All the vials were kept inside the hot air oven at 60 ºC for 7–8 h to completely dry the biomass. The weight of the vials with the dried biomass was recorded. The weights of the vials, pre- and post-drying, were used to calculate the dry weight of the obtained biomass^22^.

### Lipid analysis

Sulpho-phospho vanillin (SPV) method was used for lipid estimation. 10 mg of dry biomass was added to 2 mL of 98% sulphuric acid in a vial. It was vortexed and incubated in a hot water bath at 100 °C for 10 min. The solution was allowed to cool down to room temperature and 5 mL of phosphovanillin reagent was added. Phosphovanillin was previously prepared by adding 120 mg of vanillin in 80 mL of 85% ortho-phosphoric acid and 20 mL of ethanol (90%). The solution was vortexed and incubated at 37 °C, 200 rpm for 20 min. A color change was observed, and optical density was recorded at 530 nm^23^.

### Synthesis and characterization of CaO bionanocatalyst

The residual microalgal biomass obtained after lipid extraction was dried and used to prepare the heterogeneous catalyst. The biomass and calcium nitrate were used for the synthesis of CaO nanocatalyst. The procedure began with the heating of biomass in distilled water at 90 °C for 1 h. The resulting solution was then filtered out using filter paper and mixed with 0.5 M calcium nitrate solution (1:9 v/v). 1 N NaOH was added dropwise till complete precipitates were formed. The pH was neutralized by washing it with distilled water. The obtained pellet was calcinated at 800 °C for 2 h^16^. Characterization was carried out using scanning electron microscopy (SEM); energy dispersive X-ray (EDX) analysis was performed using ZEISS EVO MA18 with varied pressure modes at a 20 kV working voltage, Fourier transform infrared spectroscopy (FT-IR), was performed using a Shimadzu-8400 S spectrometer. X-ray diffraction (XRD) spectra were recorded using a Rigaku X-ray diffractometer. The crystalline particle size was calculated using the Scherrer equation^24^.

### Transesterification

For the transesterification reaction, the lipid was first extracted using the modified Bligh and Dyer method, a gravimetric approach^14^. 4 mg of the extracted lipid was taken in a clean dry glass ampoule and dissolved in 1 mL chloroform. To this mixture, 850 µL of methanol and 0.2 mg of synthesized CaO bionanocatalyst were added. The ampoule was sealed and incubated in a hot water bath for 4 h at 90º C. After incubation the seal was broken and 500 µL of double distilled water was added to it. It was left for phase separation for 2–5 min after which the lower phase was collected for ^1^H NMR and ^13^C NMR analyses to check for the presence of methyl esters^16^. Bruker spectrometer was used to record both types of spectra with chloroform and CDCl_3_, used as an internal reference and solvent, respectively. GC-MS was performed using Agilent GCTQ7000E to confirm the presence of methyl esters and determine the components FAMEs in the sample.

## Results and discussion

### Biochemical characterization

The biochemical composition of MO was determined to quantify the number of proteins, carbohydrates, and pigments (chlorophylls a, b, and total chlorophyll). The protein and carbohydrate content were found to be 14.17 ± 0.008% and 46.77 ± 0.0008% (w/w). Average protein and carbohydrate content has been found to vary between 10 and 50% and 2–30% in different microalgal species. *Dunaliella* sp., *Chlorella* sp., *and Scenedesmus* sp. have proteins ranging between 11 and 18%, 50–60%, and 20–45%, respectively^20^. In a study, *Monoraphidium contortum* showed 12.84% protein content whereas the carbohydrate content was found to be 5.95%^21^. The carbohydrate content in *D. salina*, *Haematococcus pluvialis*,* S. obliquus*,* and Porphyridium cruentum* was reported to be 32%, 27%, 40%, and 40–57% respectively. Further in the study, chlorophyll a, chlorophyll b, and total chlorophyll was found to be 1.218 ± 0.05, 6.86 ± 0.03, and 7.96 ± 0.07 mg/g. Similar results were reported for *Monoraphidium braunii* where chlorophyll a and b was found to be > 1 and < 0.5 mg/g respectively^25^. Microalgal species such as *D. salina* ABRIINW-I1 reported higher concentrations of chlorophyll a as compared to chlorophyll b^20^. In another study for *M. contortum* chlorophyll a and b were found to be > 5 mg/g and > 2 mg/g, respectively. For *M. griffithii* and *M. litorale*, chlorophyll a and b were found to vary between 10 and 15 mg/g and 2–4 mg/g respectively^21^.

### Optimization of growth of parameters

**Fig. 2:**
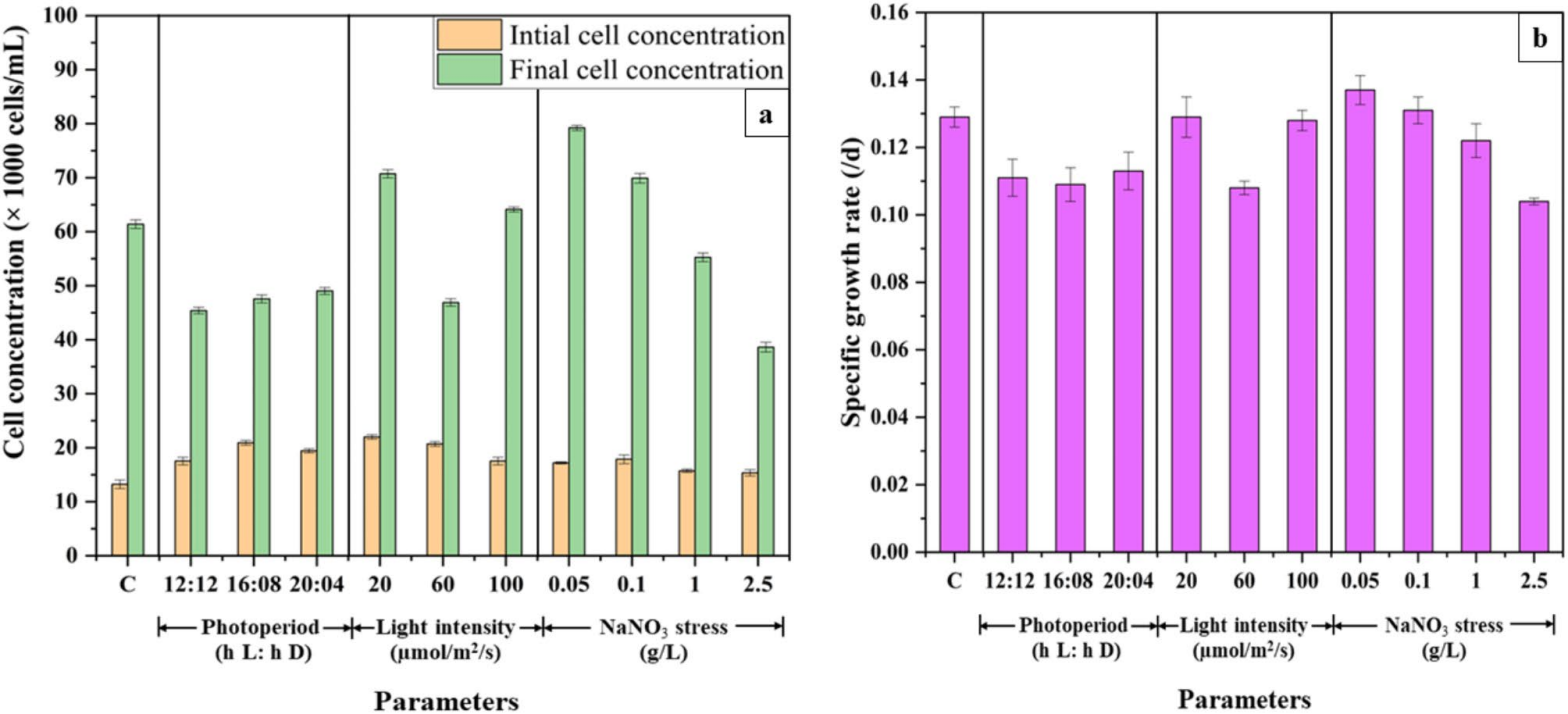
(**a**) cell concentration on initial (0th ) and final (30th ) days (**b**) Specific growth rates of MO under different growth conditions (*n* = 3).

The three selected physical growth parameters, viz., photoperiod (h L: h D), light intensity (µmol/m^2^/s), and nitrogen concentration (g/L of NaNO_3_) show significant effects on the growth and lipid production by microalgae. The effect of physical parameters on cell concentration and specific growth rate varies from species to species (Fig. 2a and b).

#### Effect of photoperiod

To study the effect of photoperiod on the growth and lipid production profile of MO, the cycle (h L: h D) was varied as 10:14 (for the control), 12:12, 16:08, and 20:04. The other two parameters viz., light intensity and NaNO_3_ concentration (N-source) were fixed at 40 µmol/m^2^/s and 0.25 g/L.

The optimum cell growth of MO was found at photoperiods of 12:12 and 10:14 with a specific growth rate of 0.111 /d and 0.129 /d, respectively. There is a significant increase in the number of cells from day 0 to day 30 of cultivation under each photoperiod regime (Fig. 2b). For the photoperiods 12:12, 16:08 and 20:04, the cell concentration was recorded to be 45.4 × 10^3^, 47.56 × 10^3^ and 49.03 × 10^3^ cells/mL respectively, on 30th day (Fig. 2a). 16:08 and 20:04 showed no significant difference, exhibiting specific growth rates of 0.109 /d and 0.113 /d respectively (Fig. 2b). However, the best cell growth results were obtained for 10:14 (control) where the final cell concentration of 61.4 × 10^3^ cells/mL was observed. For *Nannochloropsis* sp., the highest cell concentration of 6.5 × 10^7^ cells/mL, and for *Verrucodesmus verrucosus*, 6 × 10^6^ cells/mL have been reported at 16:08^26,27^. *Neochloris* sp. shows highest cell growth at 12:12^28^. In addition, it has been found that longer photoperiod increases cell concentration in *Chlorococcum* sp., however, extreme photoperiod duration viz., 24:0 exhibits lower cell concentration^29^. It has been reported that for a prolonged growth of microalgae, the photoperiods of 10:14 and 12:12 are ideal^26^. Similar results were found in the current study also where MO cultivated at 10:14 and 12:12 were more viable as compared to that at 16:08 and 20:04.

The color difference was also visually observed in some of the flasks, owing to pigment reduction in microalgal cells. This phenomenon of photobleaching is directly linked to inhibited cellular growth but there is no direct relation between photobleaching and lipid accumulation. For MO, the photobleaching effect was observed for 20:04 and lesser for 16:08. Under stress conditions, photosynthesis, and cell growth are restricted leading to nutrient deficiency which further causes yellowing, chlorosis, retarded growth, and reduced levels of pigments such as chlorophyll in microalgae^30^. Photobleaching occurs because of reduced levels of pigments and nutrients. Decreased level of mineral content in microalgae alters cell permeability, stomatal regulation, and membrane composition which directly influences its growth and metabolism^31^. Photobleaching or pigment bleaching is a condition that indicates disrupted electron transport systems as well as photosystems I and II^10^.

**Fig. 3:**
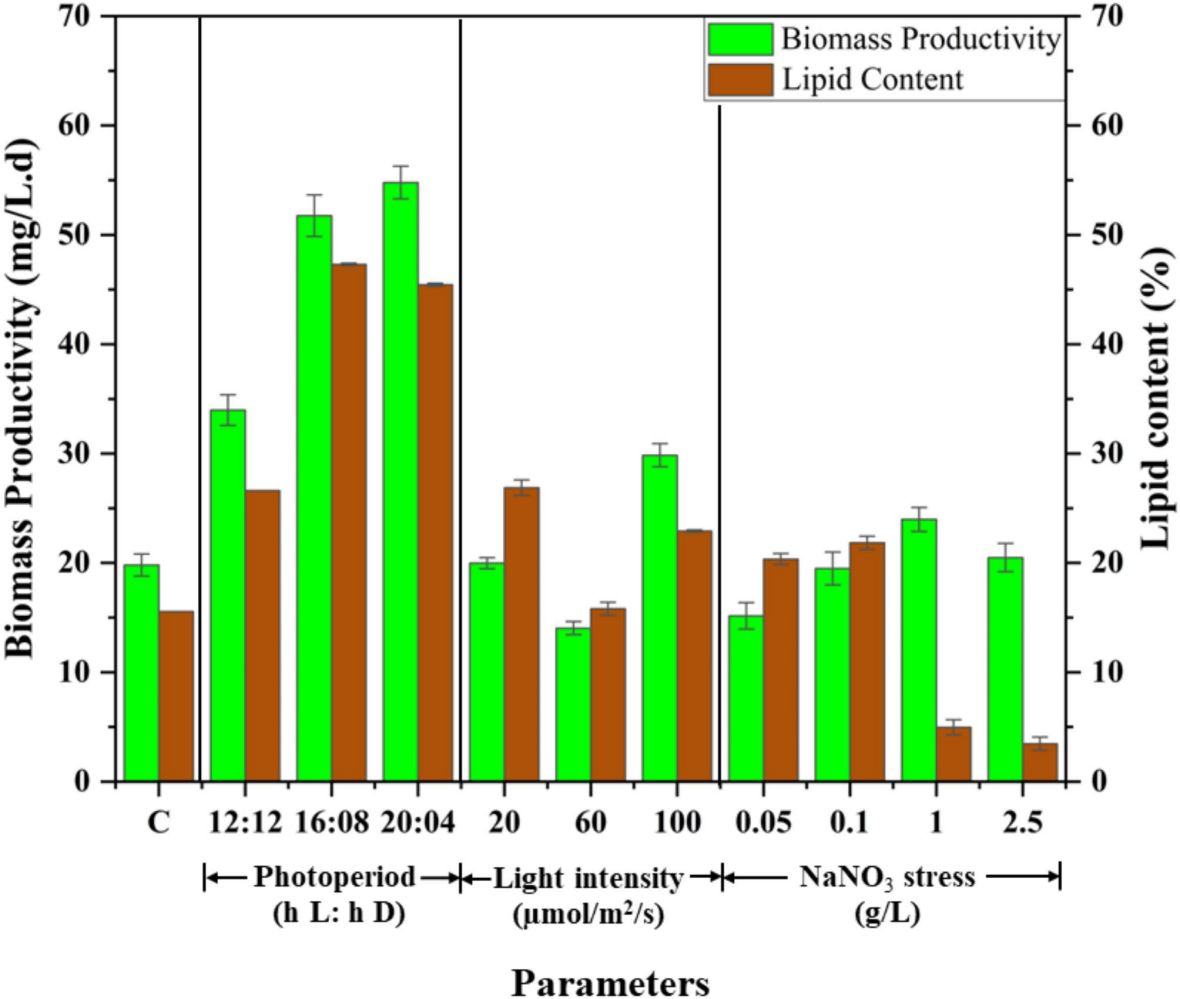
Comparative analysis of biomass and lipid content from MO, with variation in growth parameters (Control: 10 h L:14 h D, 40 µmol/m^2^/s and 0.25 g/L NaNO_3_).

An increase in biomass productivity was observed with increased duration of light exposure. At 20:04, the biomass productivity was found to be 54.79 ± 2.5 mg/L.d, whereas at 16:08, it was 51.75 ± 1.9 mg/L.d (Fig. 3). In another study on optimization of photoperiod for *C. vulgaris* it has been shown that biomass productivity increases when the cycle is flipped to 16 h L: 08 h D from 08 h L: 16 h D^32^. For *Nannochloropsis* sp., increased biomass productivity was found at 12 h and 18 h light exposure, signifying prolonged light exposure increases the growth of microalgae^26^. In the current study, biomass productivity increased with an increase in the duration of light exposure. It has been found that an increased rate of cell growth and biomass productivity is attributed to increased photosynthetic and reproduction rates. Once the cells reach their saturation limit, the growth rate becomes static and cells undergo morphological modifications to store secondary metabolites^33^.

As regards the effect on lipid productivity, prolonged exposure to light induces stress in the microalgae leading to the production of lipids, viz., the secondary metabolites. For MO, both the photoperiods 16:08 and 20:04 showed nearly similar lipid contents of 47.3 ± 2% (w/w) and 45.43 ± 4.1% (w/w), respectively. It was higher than the lipid content obtained at 12:12 and 10:14, viz., 15.55% (w/w) and 26.62% (w/w) respectively (Fig. 3). Wahidin et al. (2013), found a lipid content of 31.3% (w/w) at 16:08 for *Nannochloropsis* sp., at the end of 8th day. The productivity decreased at 12:12 to 25.59% (w/w) and at 24:0 to 27.95% (w/w)^26^. A similar trend was observed in the current study for MO wherein the lipid content decreased with reduced photoperiods (12:12 and 10:14) as well as an extended photoperiod (20:04). The current study reports an increase in lipid content at 16:08 at the end of 30th day of cultivation. Once the microalgal cell reaches its saturation limit, its growth is retarded due to reduced level of nutrient uptake and disrupted photosystem I and II. This eventually results in cell death and thereby the level of secondary metabolites is also reduced^10^. Few studies reported that a photoperiod of 12:12 is ideal for lipid accumulation in certain microalgal species such as *V. verrucosus*, and *B. braunii* signifying the varied effect of photoperiod, from species to species^27,34^.

#### Effect of light intensity

The variation in light intensity incident on the cultures was another parameter chosen to study the effect on biomass and lipid productivity. Accordingly, the control flask had 40 µmol/m^2^/s light exposure. The experimental study set had 20, 60, and 100 µmol/m^2^/s. For all the variations, the photoperiod and NaNO_3_ concentration (N-source) were fixed at 10 h L: 14 h D and 0.25 g/L.

The final cell concentration recorded for 20, 40, 60 and 100 µmol/m^2^/s were 70.73 × 10^3^, 61.4 × 10^3^, 46.9 × 10^3^, and 64.13 × 10^3^ cells/mL, respectively (Fig. 2a). The cell concentration decreased until 60 µmol/m^2^/s and increased at 100 µmol/m^2^/s. The specific growth rate was recorded highest at 20 and 100 µmol/m^2^/s, viz., 0.129 /d and 0.128 /d, respectively (Fig. 2b). It has been reported that cell growth increases with increasing light intensity for *Chlorococcum* sp. However, in another study, it was observed that both low and high light intensity of 29.41 and 44.12 µmol/m^2^/s showed the highest cell concentration, whereas the cell concentration was reduced in between the range^29^. Exposure to constant high-intensity light increases the temperature of the culture medium which affects microalgal growth. Such exposure may also affect the cellular morphology^35^. It disrupts the photosystems and causes photobleaching. Increased lipid content in the early growth phase of microalgae may be attributed to the preliminary acclimatization stress.

In the experimental runs studying the effect of light intensity on biomass productivity, a significant growth and color change was observed in all the flasks, however, a slight photobleaching effect was observed where the light intensity was 100 µmol/m^2^/s. It was found that the flask exposed to 100 µmol/m^2^/s light intensity showed the highest biomass productivity for MO at 29.85 ± 1.06 mg/L.d. (Fig. 3). Similar results were observed for *Ankistrodesmus falcatus*,* C. emersonii*,* Chaetoceros muelleri*,* and I. galbana* where the biomass yield proportionally rose with light intensity. In that study, the highest biomass productivity was observed at 135 µmol/m^2^/s^7^. For MO, the biomass productivity at 20 and 40 µmol/m^2^/s *viz*., 19.98 ± 0.5 and 19.81 ± 1.01 mg/L.d, did not show any significant difference. Further, with an increase in light intensity, viz., 60 µmol/m^2^/s the biomass productivity decreased (14.04 ± 0.6 mg/L.d) indicating saturation in microalgae, viz., photoinhibition^7^. At this point, all saturated microalgal cells undergo the death phase, and therefore a reduction in biomass yield is observed. However, on further being exposed to higher light intensity the microalgae undergo photo-acclimation, a process that promotes growth and photosynthetic activity^36^. Therefore, a significant increase in biomass productivity was observed at 100 µmol/m^2^/s. Prolonged exposure to this intensity light induces stress in microalgae leading to increased lipid content.

In the study to observe the effect on lipid productivity, high lipid content was obtained at 20 and 100 µmol/m^2^/s., viz., the lowest and highest light intensities that were selected. At 20 µmol/m^2^/s the lipid content for MO was found to be 26.88 ± 2.1% (w/w) (Fig. 3). In another study at the same light intensity for *Chlorella* sp., the lipid content was found to be nearly the same, viz., 26.94% (w/w)^37^. It has been found that between intense light intensity and periodic light and darkness, exposure to low light intensity for a fixed period significantly increases lipid productivity^26^. Light intensity study on *Ettlia* sp., showed that extreme low and high light intensity reduced the growth and lipid productivity^38^. At 40 (control) and 60 µmol/m^2^/s the lipid content decreased which may be because of reduced levels of stress. Many tudies have reported 40 and 60 µmol/m^2^/s to be optimum light intensities for the growth of microalgal species, such as *Scenedesmus* sp., *C. vulgaris*^7,39^. Hence, no stress is induced in the microalgae for enhanced lipid accumulation. Further, 20 and 100 µmol/m^2^/s are extremely low and high light intensities; thus, they induce stress in the microalgae. In this study, the lipid content started increasing from 60 µmol/m^2^/s to 100 µmol/m^2^/s. At 100 µmol/m^2^/s, 22.92 ± 1.2% (w/w) lipid content was observed for MO lower than 20 µmol/m^2^/s because of photo-acclimation. Similar results have been found for species such as *I. galbana*,* Koliella antartica*,* Botryococcus braunii*,* Chlorococcum oleofaciens*^40,41^. Prolonged exposure to high-intensity light induces stress in microalgae and increases cell size which is ascribed to the synthesis of various secondary metabolites and lipids. Thus, even if lipid accumulation is high under stress conditions, the microalgae would soon reach the death phase. Therefore, in this case for MO, a light intensity of 20 µmol/m^2^/s was selected as the optimum one.

#### Effect of nitrogen stress

In the last batch of experimental studies, MO was subjected to nitrogen stress by way of varying the NaNO_3_ concentration in BBM medium, keeping all the other nutrient composition unchanged. The concentration of NaNO_3_ was varied as 0.05, 0.1, 0.25 (control), 1 and 2.5 g/L. In all the variations, the photoperiod and light intensity were fixed at 10 h L: 14 h D and 40 µmol/m^2^/s.

Post 30 days of inoculation, a significant color change was observed in all the flasks. The cell concentration decreased with nitrogen enrichment in the growth medium. The highest final cell concentration of 79.23 × 10^3^ cells/mL was observed at 0.05 g/L followed by 69.9 × 10^3^ cells/mL at 0.1 g/L of nitrogen concentration (Fig. 2a). The specific growth rates for various nitrogen concentrations, viz., 0.05, 0.1, 0.25, 1 and 2.5 g/L were found to be 0.137, 0.131, 0.129, 0.122 and 0.104 /d, respectively (Fig. 2b). Nitrogen promotes cellular growth and metabolism as it is one the key elements involved in cell growth^42^. However, it has been reported that excessive nitrogen concentration reduces the cell growth and cell density due to nitrogen toxicity^43^. Though nitrogen is a crucial element for cell growth and metabolism it has been found that each species has its own saturation limit for nitrogen assimilation. Thus, nitrogen supplementation depending on the type of species shows different responses, viz., either promotes growth or induces stress further leading to ammonia toxicity and eventually cell death. Mainly nitrogen limitation or enrichment induces stress in microalgae which results in the production of secondary metabolites and thus biomass increases. Prolonged exposure to nitrogen-enriched medium alters the metabolic pathway in microalgae, thus both cell concentration and biomass productivity are reduced in later stages^44^.

It was observed that with the increase in nitrogen concentration, the biomass yields increased. The highest biomass yield was found at 1 g/L NaNO_3_ concentration and the lowest was found at 0.05 g/L, viz., 23.98 ± 2.1 mg/L.d and 15.16 ± 1.2 mg/L.d (Fig. 3). Nitrogen deprivation inhibited cell growth and thus biomass yield decreased from 1 g/L to 0.05 g/L^42^. For two microalgal species namely, *A. falcatus* and *C. emersonii*, a similar trend was observed where the biomass yield increased with increased N- concentration (0–10 g/L). However, in the same study, it was found that for *C. muelleri* and *I. galbana* the biomass yield increased at 5 g/L and then decreased at 10 g/L^7^. For MO, the biomass yield increased up to 1 g/L and later decreased with increasing the NaNO_3_ concentration to 2.5 g/L (Fig. 3). A study on the nitrate metabolism pathway for microalgae suggests that the breakdown of nitrate, formation of nitrite and ammonia are linked to each other^44^. Excessive nitrogen uptake results in the accumulation of ammonia which has been proven to be toxic to microalgal growth^45^. Excessive nitrogen concentration changes the pH of the culture medium and reduces microalgal growth and development^43^.

At 0.1 g/L and 0.05 g/L NaNO_3_ concentration, a high lipid content of 21.84% (w/w) and 20.34% (w/w) were observed respectively. Similar results were found for *Tetraselmis* sp., where lipid content > 20% was reported^46^. The lipid productivity decreased with further enrichment of NaNO_3_, viz., 0.25, 1, and 2.5 g/L in the growth medium (Fig. 3). Nitrogen stress, i.e., reducing the nitrogen concentration, has proved to induce more stress as compared to nitrogen-enriched conditions^42^. NaNO_3_ enrichment in the growth medium promotes cell growth, cellular metabolism, and photosynthetic activity, thus the formation of secondary metabolites is reduced. Whereas, under nitrogen deprivation cell growth is constrained, and cell size starts increasing which ascribes to the formation of secondary metabolites such as lipids^42,47^. There is a shift in a metabolic pathway, viz., diacylglycerol acyltransferase is activated that produces triacylglycerols (TAGs) from fatty acid acyl-CoA^48^. This increases the neutral lipid accumulation in microalgal cells^9^. However, decreasing NaNO_3_ concentration from 0.1 to 0.05 g/L reduced the lipid content for MO. Chin et al.. (2023), studied the effect of nitrogen on four tropical microalgae, namely, *A. falcatus*, *C. emersonii*,* C. muelleri* and *I. galbana* under three concentrations, 0, 5, and 10 g/L of NaNO_3_. Similar results were observed where optimum lipid productivity was found at 5 g/L and both 0 and 10 g/L comparatively showed less productivity^7^. The process of nitrogen starvation occurs in two ways (1) sudden starvation and (2) progressive starvation. Sudden starvation refers to the maximum removal of nitrate traces from the microalgae and then cultivating it without nitrogen in the medium, whereas in progressive starvation microalgae is cultivated with increasingly low concentrations of nitrogen in the medium^47^. The current study shows progressive starvation, therefore, reduced growth and lipid accumulation are a result of natural nitrogen shortage^7^.

A comparison of the biomass productivity and lipid yield obtained from other diverse microalgal species has been tabulated including the values obtained in the current experimental study (Table 1).

**Table 1:** Biomass productivity and lipid yield from varied microalgal feedstocks.

Species	Growth conditions				Biomass Productivity (mg/L.d)	Lipid Yield (% w/w)	References
	Growth medium	Temperature (ºC)	Light intensity (µmol/m^2^/s)	Photoperiod (h L: h D)			
*Chlorella* SDEC-4	BG-11	25 ± 1	40	24:00	8.73	19.40	^39^
*Scenedesmus* SDEC-9					4.70	30.70	
*Selenastrum* sp. GA66	N-11	25 ± 2	75	14:10	16.25	33.72	^12^
*Myrmecia bisecta*	BBM	24 ± 2	117.65	–	47.91 ± 1.07	12.68 ± 3.33	^21^
*Monoraphidium griffithii*					22.78 ± 1.2	14.27 ± 1.21	
*Oocystis lacustris*					33.63 ± 0.83	16.49 ± 1.30	
*Coelastrella* sp. M-60	BG-11	25	22.06	12:12	31.6 ± 1.5	≈ 24	^49^
*Micractinium* sp. M-13					23.3 ± 0.9	≈ 26	
*Monoraphidium* NCIM 5585	BBM	25–28	40	16:08	51.75 ± 1.9	47.3 ± 0.02	Current study

### Synthesis and characterization of CaO bionanocatalyst

**Fig. 4:**
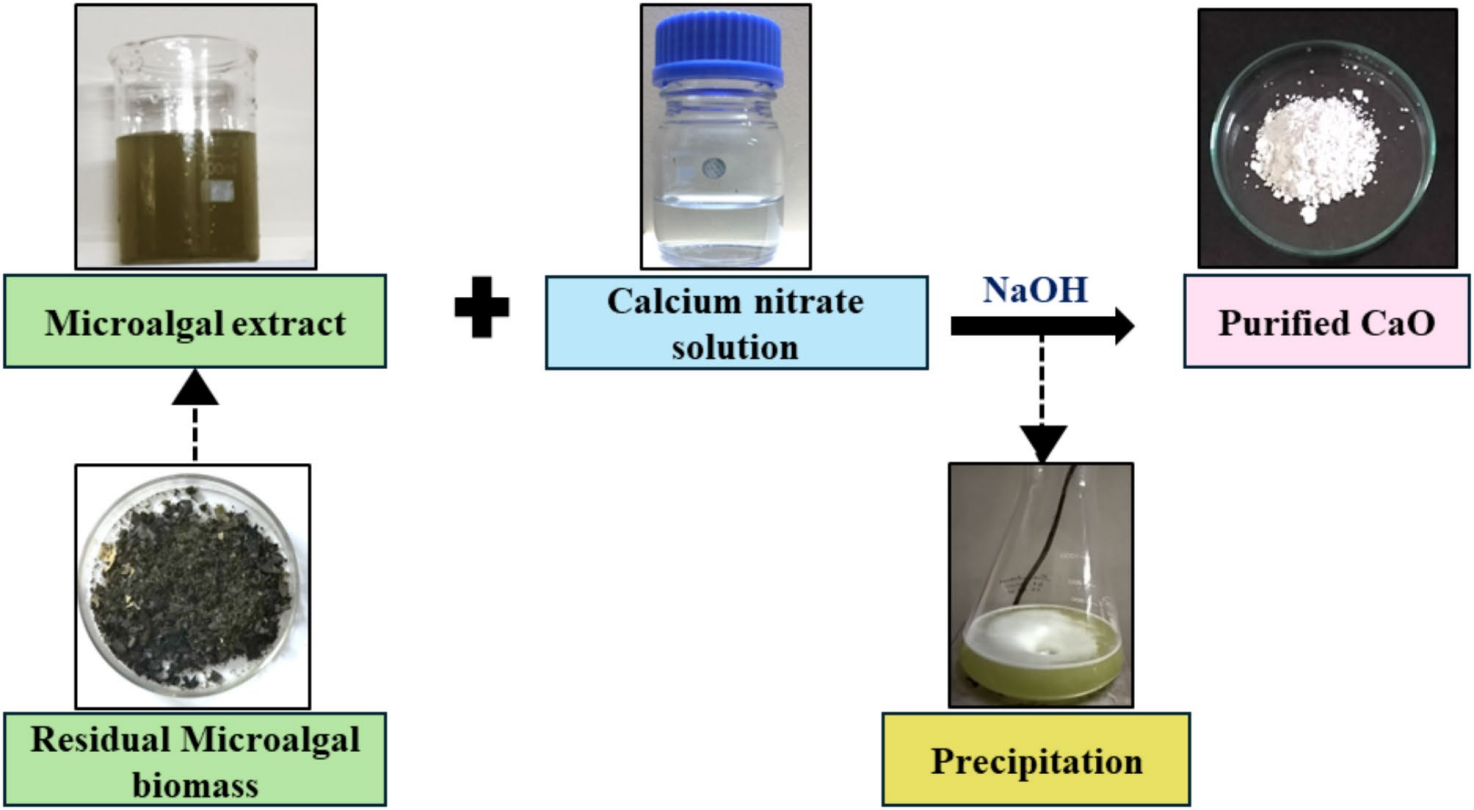
Synthesis of CaO bionanocatalyst.

Literature suggests that microalgae such as *(A) dimorphus*, *C. vulgaris*, and *(B) braunii* have been reported to be used for fabricating bionanocatalysts containing silver and silicon as nanoparticles^15^. Other macroalgal species such as *Ulva lactuca* and *Sargassum plagiophyllum* have been used in combination with silver nanoparticles. Synthesis of CaO nanoparticles has been reported using various biological extracts such as *Moringa oleifera*, *Citrullus colocynthis*, watermelon peel, and chicken eggshell^17^. In this study, a novel approach was followed where the residual microalgal biomass obtained post-lipid extraction of MO biomass was used in combination with calcium nitrate for the green synthesis of CaO bionanocatalyst (Fig. 4). The pale green microalgal extract was added to the colorless calcium nitrate solution which resulted in a shift in the solution’s color to a milky white suspension. Further heating and stirring with the addition of NaOH enhanced the precipitate formation. The catalyst was ground into a powder after several rounds of washing and drying (Fig. 4). SEM, EDX, XRD, and FT-IR were used to confirm the CaO bio nanocatalyst’s production following visual inspection.

**Fig. 5:**
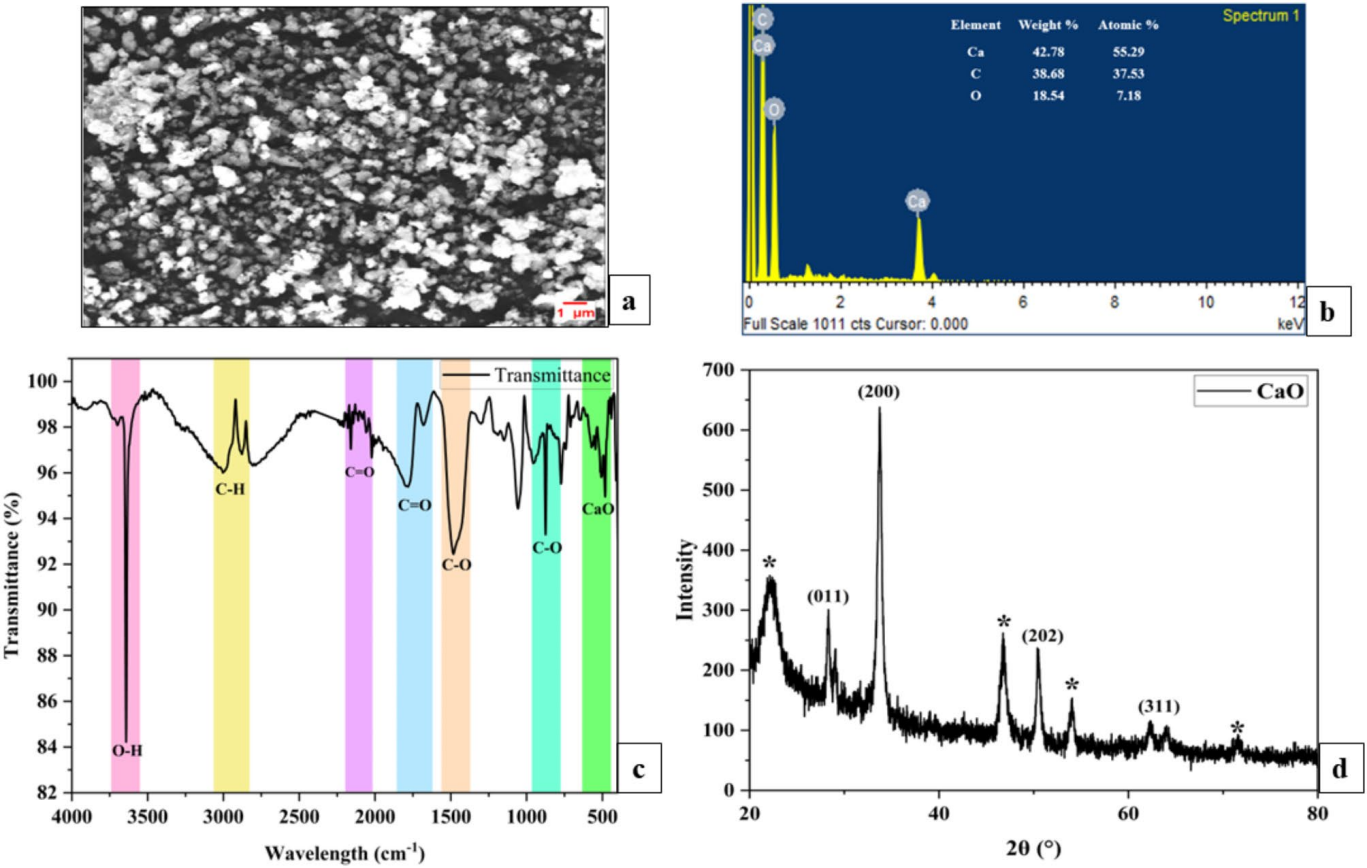
Characterization of CaO bionanocatalyst using (a) SEM (b) EDX (c) FT-IR (d) XRD.

Scanning electron microscope (SEM) analysis was carried out to determine the structural morphology of the synthesized catalyst (Fig. 5a). The results showed that the synthesized catalyst had distorted spherical shapes as the particles agglomerated and formed clusters^16^. The agglomerations observed are probably because of the capping due to phenolic compounds in the algal extract^50^. The particle size was calculated using Image J software and was found to be 39.92 nm^24^. Energy dispersive X-ray (EDX) spectroscopy was carried out to determine the composition of the CaO catalyst (Fig. 5b). Three elements were found: calcium (Ca), oxygen (O), and carbon (C) (Fig. 5b). The results were comparable with the existing literature and thus the formation of CaO-nanocatalyst was confirmed^24,51^. Signals for C were detected due to the presence of phenolics in the microalgal extract. The other weak signals obtained could signify the presence of other microalgal phytocomponents^50^.

Fourier-transform infrared (FT-IR) spectroscopy of the bionanocatalyst showed spectra in the range of 400–4000 cm^− 1^ (Fig. 5c). A sharp band was observed at 3642 cm^− 1^ which attributed to –OH vibrations that were generated due to physisorption of H_2_O on CaO surface^52^. Bands between 2200 and 1500 cm^− 1^ indicate C = O vibrations due to the presence of CaCO_3_^52,53^. Characteristic bands were observed at 1493 and 877 cm^− 1^ indicating C–O stretching and bending of CaCO_3_^16^. Further, CaO bands were observed between 544.74 and 483.42 cm^− 1^^52^. The bands observed between 3200 and 2800 cm^− 1^ contribute to C–H stretching. These additional bands were generated due to the adsorption of phytochemicals on the CaO surface^54^.

X-ray diffraction (XRD) spectrum for the bionanocatalyst showed characteristic peaks at 2θ (°) of 29.06 (0 1 1), 33.70 (2 0 0), 54.06 (2 0 2) and 63.55 (3 1 1) (Fig. 5d). Similar results were obtained for CaO catalyst synthesized from eggshell. The results were compared with JCPDS – 77–9574^16^. Peaks obtained 2θ (°) of 17.66, 22.14, and 28.32 correspond to CaCO_3_ whereas the 46.78 and 50.43 peaks signify Ca(OH)_2_^51^. Additional peaks observed in the spectrum might be due to the crystallization of compounds present in the algal extract^50^. The average crystalline size was calculated using the Debye-Scherrer equation, and it was found to be 17.83 nm.

### Transesterification

**Fig. 6:**
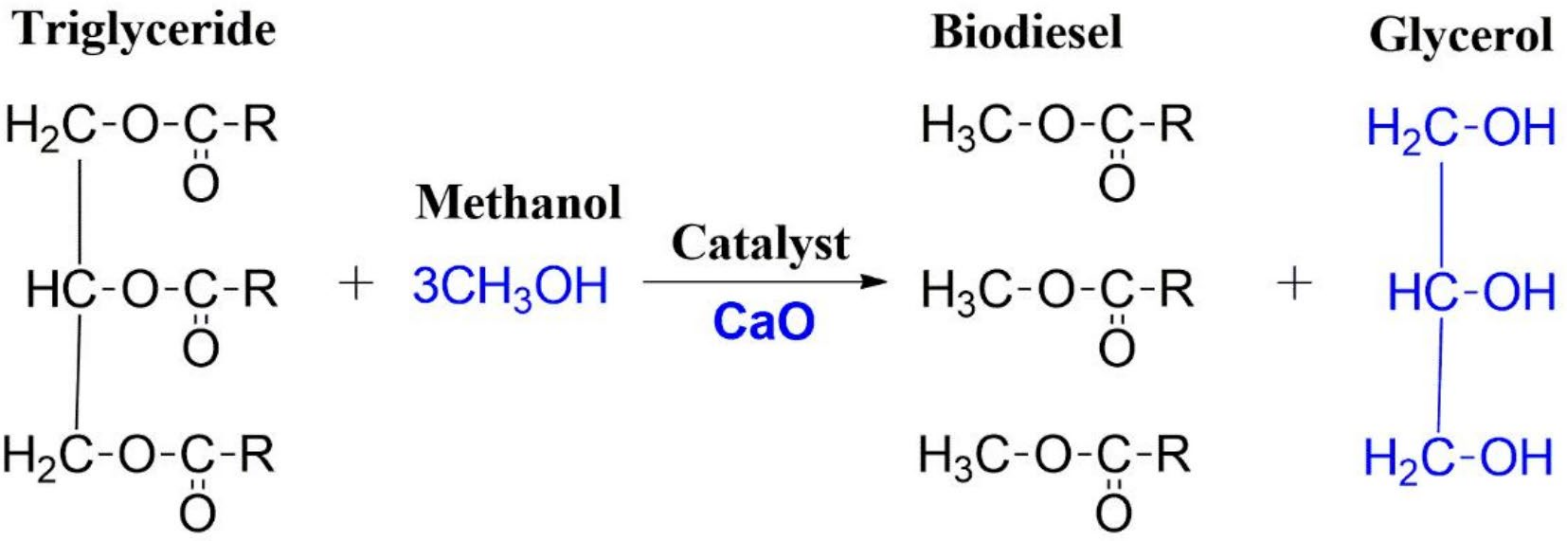
Mechanism of transesterification process.

The synthesized CaO bionanocatalyst was checked for its transesterification ability on the lipid obtained from the modified Bligh and Dyer method. The catalyst initially binds with alcohol, which subsequently reacts with triglycerides to produce fatty acid alkyl esters (FAMEs) (Fig. 6). Triglycerides are first converted into diglycerides, which further break down into monoglycerides. These monoglycerides then react with methanol to form FAMEs^16^. Post the transesterification reaction, two distinct phases were observed (1) the upper layer consisting of glycerol and water (2) Biodiesel (FAMEs). As the amount of glycerol byproduct was less to be separated directly from the FAMEs-glycerol mixture, water was added to separate glycerol. Glycerol, being soluble in water, forms a distinctive upper layer and the FAMEs constitute the bottom layer. FAME yield was found to be 86.5 ± 0.65% (w/w). Similar results of FAME yield were observed from *Acutodesmus obliquus*, where the highest biodiesel yield of 86.14% was recorded for a reaction time of 3.5 h and 1.6% catalyst load^16^. It has been reported that CaO shows better transesterification efficiency as compared to conventional catalysts. For *C. vulgaris*, *a* FAME yield of ≈ 67% and < 40% have been observed using CaO and H_2_SO_4_, respectively^55^.

**Fig. 7:**
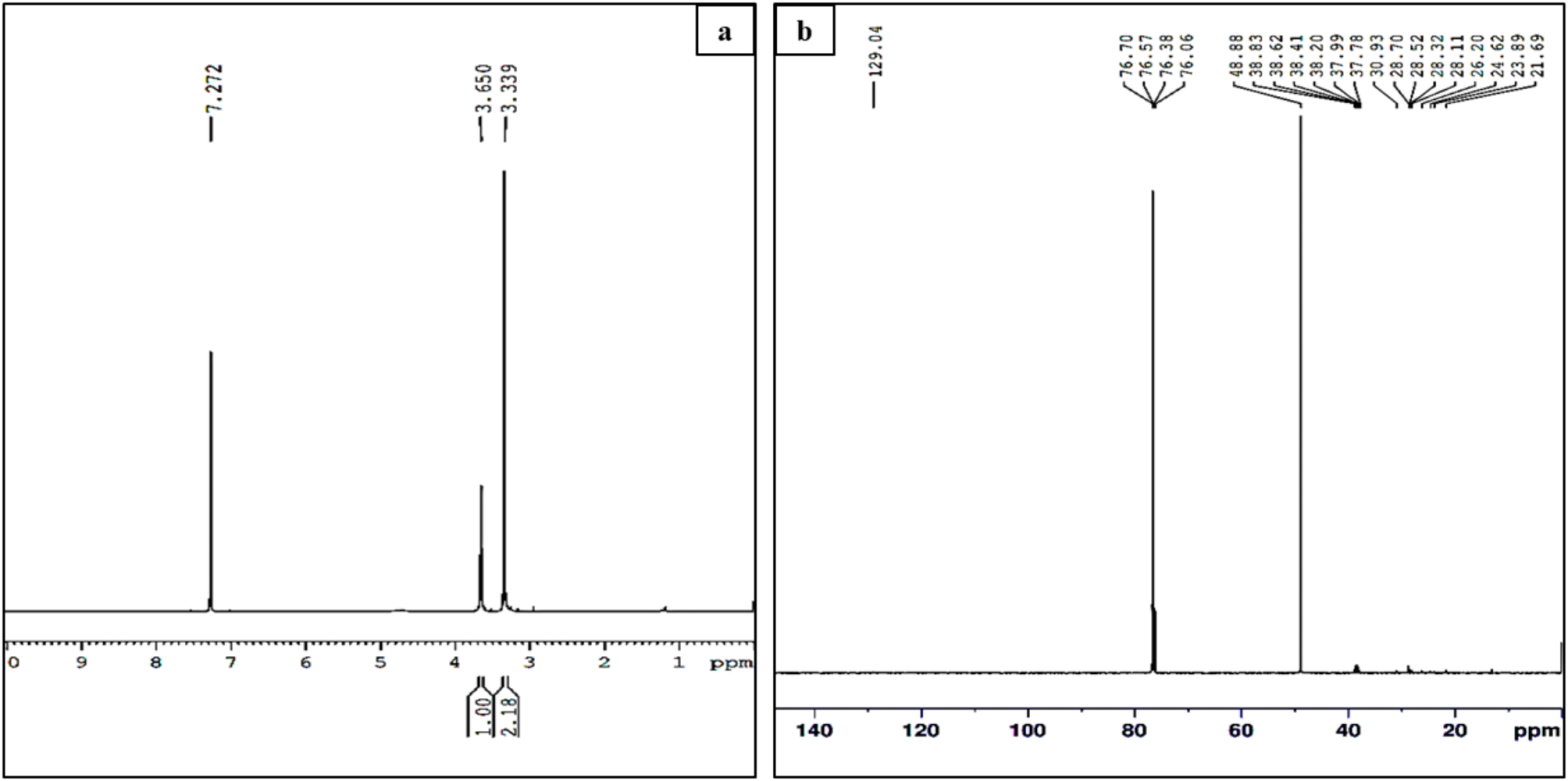
(**a**) ^1^H NMR and (**b**) ^13^C NMR of FAMEs obtained after transesterification of MO-lipid.

The FAME analysis was carried out using ^1^H NMR and two characteristic peaks were observed at 3.650 ppm and 3.339 ppm (Fig. 7a). The peak at 3.650 ppm signifies algal biodiesel consisting of methoxy proton (OCH_3_) in the methyl ester. Similar, results have been observed for biodiesel obtained from *Scenedesmus* sp., *Tetraselmis* G4, and commercial biodiesel^56,57^. It signals the presence of OCH_2_ and CH-OCH ester groups. The peak at 3.339 ppm corresponds to the ester bond, viz., CH_3_COO-CH, and is comparable with results obtained for *C. variabilis*^58^. Low-intensity peaks at 2.90 ppm and 1.2 ppm correspond to the presence of allylic and aliphatic groups respectively^56^. Another sharp peak at 7.272 ppm corresponds to the aromatic hydrogen^59^.

Further ^13^C NMR confirmed the presence of FAMEs (Fig. 7b). The signal observed at 13.07 ppm was recorded due to the terminal carbon of the methyl group. The spectrum obtained between 21.69 and 30.93 ppm ascribes to the presence of CH_2_-CH_2_ group of FAMEs. Similar results were observed in FAMEs obtained from *S. quadricauda*^60^. Signals at 21.69, 23.89–28.70, and 30.93 ppm refer to β-CH_2_, -CH_2_, and (-CH_2_)_n,_ respectively^61^. The signals at 37.78, 37.99, 38.20, 38.41, 38.62, and 38.83 ppm signify the presence of R- CH_2_- Cl group. The 48.88 ppm sharp peak was noticed due to the presence of CH_3_CO-R. This result was comparable with FAMEs obtained from *Spirulina* sp., *Chlorella* sp., and *Tetraselmis* sp^57^. Sharp peaks observed at 76.06, 76.38, 76.57, and 76.70 ppm are probably due to the presence of unsaturated alkynes. Similar results were obtained for FAMEs derived from *S. quadriquada*^60^. Therefore, the results obtained from both ^1^H and ^13^C NMR confirmed the transesterification of lipids obtained from MO to biodiesel.

**Fig. 8:**
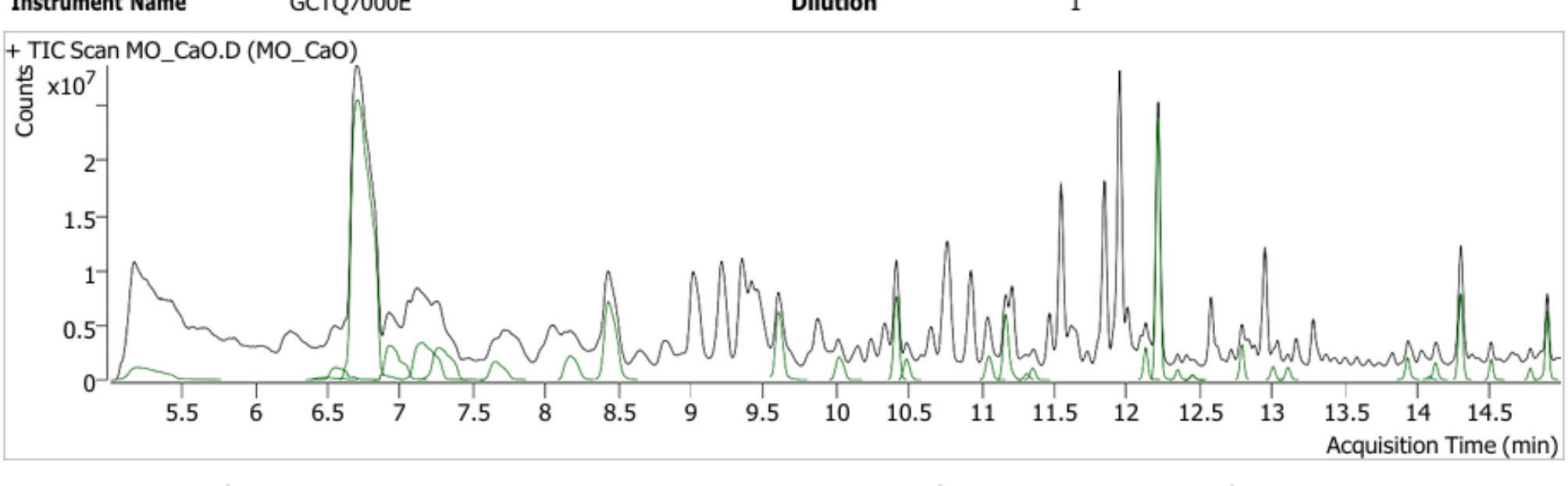
Gas Chromatogram of FAMEs obtained from MO.

Further, the composition of the obtained FAMEs was determined using GC-MS (Fig. 8). The analysis started at 70 ºC, raised to 200 ºC at a rate of 10 ºC/min, and further increased to 240 ºC at 15 ºC/min. The chromatogram consisted of significant peaks for hexanoic acid, 4-oxo-, methyl ester; dodecanoic acid, methyl ester and hexanedioic acid, dimethyl ester. Peaks for butanedioic acid, dimethyl ester; 2-butenoic acid, 4,4-dimethoxy-, methyl ester and other trace compounds were also detected. The obtained peaks signify the presence of FAMEs in the transesterified sample. A similar composition of FAMEs was obtained for biodiesel derived from *Ettlia* sp^38^. , . The structure and molecular weight of the detected FAMEs were determined using NIST.Lib., library data. The obtained fatty acids were found to be in the range of C6-C13 chain length. For *Ettlia* sp., the chain length varied between C10-C18^38^. FAMEs derived from species such *as D. pannonicus*, *P. kessleri*, *M. griffithii* and *A. falcatus* consisted of components with chain length varying between C6-C20. Significant peaks were observed at retention time (R_T_) of 10–14 min, similar to the current study^62^. Table 2 discusses the components of FAMEs, viz., molecular weight, formula and retention time (R_T_) for each of the components identified. Therefore, the transesterification efficiency of the synthesized CaO bionanocatalyst with 5% loading was validated. The lipid extracted from MO was successfully transesterified to FAMEs using the CaO bionanocatalyst.

**Table 2:** Components of FAMEs obtained from MO.

Components of FAME	*R*_T_	Molecular weight	Formula
Butanedioic acid, dimethyl ester	12.1284	C_6_H_10_O_4_	146.1412
2-Butenoic acid, 4,4-dimethoxy-, methyl ester	12.3480	C_7_H_12_O_4_	160.17
Hexanoic acid, 4-oxo-, methyl ester	12.7910	C_7_H_12_O_3_	144.1684
Dodecanoic acid, methyl ester	14.5050	C_13_H_26_O_2_	214.3443
Hexanedioic acid, dimethyl ester	14.7748	C_8_H_14_O_2_	174.1944

## Conclusion

The current investigation shed light on the potential of *Monoraphidium* sp. NCIM 5585 for biodiesel production. Studies conducted on growth condition optimization underscored the role of light intensity, photoperiod, and nitrogen concentration in influencing cell growth, biomass, and lipid productivity. The effects of each parameter showed varied results for MO when compared with other microalgae. In other words, it can be said the tolerance level in MO is different when compared to other microalgal species. Accordingly, a shift in cell concentration, specific growth rate, biomass, and lipid yield has been observed. The variation in growth and productivity are the results of metabolic shifts and the accumulation of secondary metabolites in MO under conditions of stress. Also, this study explains that though biomass yield and lipid accumulation are linked to cell growth, an increase in cell concentration does not symbolize enhanced biomass and lipid yield. An increase in biomass yield at low cell concentrations signifies stress and secondary metabolite accumulation. Likewise, high cell concentration and low biomass yield signify relatively lower stress leading to rapid cell growth and reduced secondary metabolite formation. Further, the residual MO biomass proved to be a potential source for the synthesis of cost-effective and sustainable CaO bionanocatalyst. The role of economical and sustainable CaO bionanocatalyst in the transesterification of microalgal lipids has been successfully established. Green synthesis of bionanocatalyst favors sustainable goals in addition to reducing the transesterification process cost. Optimization of growth condition facilitates large scale cultivation of microalgae in addition to identification of potential microalgal feedstock for biodiesel production. Overall, this study highlighted and discussed the significance of cultivation conditions, their potential effect on biodiesel production, key strategies to utilize residual microalgae and reduce the catalyst cost. To further expand the work, similar study and optimization experiments can be conducted for large scale production of microalgal biomass coupled with bioremediation and biofuel production.

## Data Availability

Data availabilityThe authors declare that the data supporting the findings of this study are available within the article.
